# Exploring factors influencing pregnant Women’s attitudes, perceived subjective norms and perceived behavior control towards male involvement in maternal services utilization: a baseline findings from a community based interventional study from Rukwa, rural Tanzania

**DOI:** 10.1186/s12884-020-03321-z

**Published:** 2020-10-19

**Authors:** Fabiola V. Moshi, Stephen M. Kibusi, Flora Fabian

**Affiliations:** 1 Department of Nursing and Midwifery, College of Health Sciences of the University of Dodoma, P.O. Box 259, Dodoma, Tanzania; 2 Department of Public Health, College of Health Sciences of the University of Dodoma, P.O Box.259, Dodoma, Tanzania; 3 Department of Biomedical Sciences, College of Health Sciences of the University of Dodoma, P.O Box.259, Dodoma, Tanzania

**Keywords:** Attitude, Subjective norms, Perceived behavior control, Male involvement, Pregnant women

## Abstract

**Background:**

Although male involvement enhances obstetric care-seeking behavior, the practice of male involvement in developing countries remains unacceptably low. Male involvement in maternal services utilization can be influenced by the attitude, subjective norm, and perceived behavior control of their female partners. Little is known about factors influencing pregnant women’s attitudes, perceived subjective norms, and perceived behavior control towards male involvement in maternal services utilization.

**Methods:**

A baseline community-based cross-sectional study whose target was pregnant women were performed from 1st June until 30th October 2017. A three-stage probability sampling technique was employed to obtain a sample of 546 pregnant women. A structured questionnaire that hinged the *Theory of Planned Behavior* was used. The questionnaire explored three main determinants of male involvement, which were: attitudes towards male involvement, perceived subjective norms towards male involvement, and perceived behavior control towards male involvement.

**Results:**

After adjusting for the confounders, factors influencing positive attitude towards male involvement were age at marriage [19 to 24 yrs.,(AOR = 1.568 at 95% CI =1.044–2.353), more than 24 yrs. (AOR = 2.15 at 95% CI = 1.150–1.159)]; education status [primary school (AOR = 1.713 at 95% CI = 1.137–2.58)] and economic status [earning more than one dollar per day (AOR = 1.547 at 95% CI = 1.026–2.332)]. Factors influencing perceived subjective norms was only age at marriage [19 to 24 yrs., (AOR = 1.447 at 95% CI = 0.970–2.159), more than 24 years, (AOR = 2.331 at 95% CI = 1.261–4.308)]; factors influencing perceived behavior control were age at marriage [more than 24 years (AOR = 2.331 at 95%CI = 1.261–4.308)], and the intention to be accompanied by their male partners (AOR = 1.827 at 95%CI = 1.171–2.849).

**Conclusion:**

The study revealed that women who were married at an older age were more likely to have a positive attitude, subjective norms, and perceived behavior control towards male involvement in maternal services utilization than those who were married at a young age. Pregnant women who had primary education and earn more than a dollar per day were more likely to have positive attitudes towards male involvement than poor and uneducated pregnant women. The study recommends an interventional study to evaluate the influence attitude, subjective norms, and perceived behavior control on male involvement in maternal services utilization.

## Background

Maternal mortality is a public health challenge worldwide. In 2015, 303,000 maternal deaths were estimated to have occurred globally [1]. Nearly all of these deaths occurred in low resources countries [1]. In Tanzania, the reckoned maternal mortality ratio was 556/100,000 [2], meaning that for every 1000 live births, about 5 women died due to pregnancy-related causes which amounted to 8000 maternal deaths per year. Therefore, Tanzania was categorized to be among the countries in Africa with the highest maternal mortalities.

Low male involvement in maternal services utilization in low resources countries has been cited as one of the factors contributing to high maternal mortality in these countries [3]. Male involvement in maternal services utilization has been expressed as a practice of social and behavioral change that is needed for men to take more responsibility in maternal services utilization with the focus of ensuring women’s and children’s health [4].

There are complex behavioral and cultural factors influencing male partner’s involvement in the care of their expecting wives/partners in Tanzania [5]. The evidence indicates that efforts that embrace male partners and uphold gender-equitable relationships between men and women are more efficient in producing behavior change than narrowly focused interventions [6].

The practice of male involvement in developing countries including Tanzania remains unacceptably low [7–9]. The previous study was done by Sokoya Masunmola et al., [10] has reported that although both men and women are in support of male involvement during pregnancy and childbirth surprisingly very few men were involved in maternal services utilization. The low male involvement practice could be rooted in cultural gender roles where pregnancy care and childbirth are believed to be women’s responsibility [3] while men’s responsibility is to provide financial support [5].

The effect of gender roles and responsibilities does matter in actual male involvement in maternal services utilization in low resources settings including Tanzania [5]. It has been a norm in rural settings that pregnant care, childbirth, and post-delivery care are solemnly responsible for women [5]. The responsibility of the male partner is to provide financial support [5]. The trend in low resources settings is now struggling to change from the traditional maternal services delivery from addressing a pregnant woman to addressing couples. If pregnant women understand and accept positively on involving their male partners in maternal services utilization, the state of male involvement in maternal services utilization will improve dramatically.

As well, pregnant women’s negative attitude towards male involvement [8] is among the barriers towards male involvement. The negative attitude is due to three aspects, the perception that pregnancy and childbirth are the responsibility of women [5, 8], avoiding negative stereotyping [8], and fear male involvement may decrease their superior power and end up being insecure like women [8].

Studies have also reported unfair reproductive health programmes for women without partners as a contributing factor for low male involvement in maternal services utilization [9]. This means the struggle to bring male partners in maternal health services utilization must go hand in hand with creating male-centered services. It should go beyond physical presence. Although the center of care is a pregnant woman, a man should feel involved by either his vital signs taken or given health education specifically for him. Tanzania is among the countries with low male involvement in maternal health services especially the rural communities [10]. There is a direct relationship between male involvement and cultural beliefs which means that the societal perception and beliefs about male involvement do affect male involvement [11]. When there is a cultural belief that disapproves of the involvement of male partners in maternal services utilization, there is low male involvement despite educational interventions and mobilizations to improve male involvement.

Male partners may be willing to learn their roles in maternal services utilization but the existing perception that pregnant care issues are solemnly responsibility of women may act as a barrier towards their involvement in maternal services utilization [12, 13]. This study invested in studying the attitudes perceived subjective norms and perceived behavior control towards male involvement in maternal services utilization among pregnant women.

According to *the Theory of Planned Behavior*, behavior intention is influenced by three predictors which are attitude, subjective norms, and perceived behavior control [14]. Attitude is influenced by individual beliefs and the evaluation of behavioral outcomes. The perceived subjective norms are the way a pregnant woman perceives her society approves of disapproves male involvement in maternal services utilization. If she perceived her society approves for her to be accompanied by her male partner, she will act in favor of male involvement but if she perceives her society disapproves of the act then she will act accordingly. The perceived behavior control is influenced by control beliefs and perceived power.

Therefore, there was a need to determine pregnant women’s attitudes, perceived subjective norms, and perceived behavior control towards male involvement in maternal services utilization. The study also went further to explore factors that are associated with attitudes, subjective norms, and perceived behavior control towards male involvement in maternal services utilization.

## Methods

### Study design and setting

It was a community-based cross-sectional study done in Rukwa Region from 1st June to 30th October 2017, among pregnant women from forty-five villages. According to the national census of 2012, Rukwa had a population of 1,004,539 people; 487,311 males, and 517,228 females. The region has the lowest mean age at a marriage where male marry at the age of 23.3 years and female marry at age of 19.9 years and has a fertility rate of 7.3 [15].

### Sampling method and sample size

#### Sampling technique

Rukwa region has four administrative districts. Two districts (Sumbawanga and Kalambo Districts) were purposively selected from the four districts due to the high proportion of home birth assisted by unskilled birth attendants [16]. Three stages of sampling technique were used to obtain study participants. In the first random samplings, a simple random sampling technique was used to obtain five wards from 12 wards of Sumbawanga district and ten wards from 17 wards of Kalambo district. In the second stage random samplings, all villages in the selected wards were listed separately from each district and a simple random sampling technique using the lottery method was used to select 15 villages from Sumbawanga rural district and thirty villages from Kalambo district. A systematic sampling technique was used in the third stage sampling. Households with pregnant women of 24 weeks gestation age or less and living with a male partner were systematically selected. The first household was randomly selected; a female partner was assessed for signs and symptoms of pregnancy. For a female partner who had missed her period for 2 months was requested to test for pregnancy. For those with positive tests and consented to participate were enrolled in the study. If a selected household had no eligible participants, the household was skipped and researchers entered into the next household in the predetermined direction.

#### Sample size calculation

The sample size was calculated using the following formula [17].

$$ \mathrm{n}=\frac{{\left\{\mathrm{Z}\upalpha \sqrt{\left[\uppi \mathrm{o}\left(1-\uppi \mathrm{o}\right)\right]}+2\upbeta \sqrt{\left[\uppi 1\left(1-\uppi 1\right)\right]}\right\}}^2}{{\left(\uppi 1-\uppi \mathrm{o}\right)}^2} $$

Where:

*n =* maximum sample size.

Ζα = Standard normal deviation (1.96) at 95% confidence level for this study.

2β = standard normal deviate (0.84) with a power of demonstrating a statistically significant difference before and after the intervention between the two groups at 90%.

πο = Proportion at pre- intervention (Use of skilled delivery in Rukwa region 30.1%) [16].

π1_=_ proportion after intervention (Proportion of families which would access skilled birth attendant 51%) [16].

$$ \mathrm{n}=\frac{{\left\{1.96\sqrt{\left[0.301\left(1-0.301\right)\right]+0.84\;\sqrt{\left[0.51\left(1-0.51\right)\right]}}\right\}}^2}{{\left(0.6-0.51\right)}^2} $$

$$ \mathrm{n}=162\ \mathrm{couples}+10\%=180 $$

The required sample size in the intervention group = 180 pregnant women.

Intervention: control ratio = 1:2. Sample size in the control group = 360 pregnant women. Therefore the total sample size was 546 pregnant women.

#### Data collection procedure

Data was collected using interviewer-administered questionnaires. The theory of planned behavior questionnaire guide was used to guide the development of the questionnaire [8]. The questionnaire was translated into Swahili and was pretested before actual administration. Four trained research assistants were recruited, trained, and participated in data collection. The tool had two parts; the social demographic characteristics and a Likert scale where respondents were supposed to strongly agree, agree, neutral, disagree, and strongly disagree. The Likert scale was subdivided into three subparts of the statements in the Likert scale which were; i) attitudes towards male involvement questions ii) perceived subjective norms towards male involvement iii) perceived behavior control towards male involvement in maternal services utilization.

Attitudes towards male involvement had five Likert scale statements which were if my husband participates in setting apart of the skilled birth attendant, he is doing a good and beneficial thing, if my husband accompanies me during antenatal clinics, he is doing a good and beneficial thing, if my husband tests for HIV with me during pregnancy, he is doing a good and beneficial thing, if my husband accompanies me during childbirth, he is doing a good thing which is beneficial and if my husband accompanies me for postnatal checkups, he is doing a good and beneficial thing. Likert scale statements involved in measuring perceived subjective norms towards male involvement were; the eminent person to me believe my husband should participate in earmarking of the skilled birth attendant, eminent person to me believe my husband to escort me during antenatal clinics, eminent person to me believe my husband has to test for HIV with me during antenatal visits, eminent person to me believe my husband has to accompany me during childbirth and eminent person to me believe my husband has to escort me during postnatal checkups. Perceived behavior control was measured using the following Likert scale statements: my husband to participate in earmarking of the skilled birth attendant is trouble-free, for me, my husband to escort me during antenatal clinics is trouble-free, for me, my husband to test for HIV/AIDS with me during antenatal visits is trouble-free, for me, my husband to accompany me during labor and childbirth is trouble-free and for me, my husband to escort me during a postnatal checkup is trouble-free.

#### Data processing and analysis

The collected data were verified for integrity then coded and entered in to computer using statistical package IBM SPSS version 23. Descriptive statistics were used to generate frequency distribution and cross-tabulation used to describe the characteristic of the study participants. Factor analysis was done to measure attitude, subjective norms, and perceived behavior control. The normality test was tested and the mean score was established. The regression score above the mean was termed as positive and below mean negative (Table 1). Logistic regression was done to determine the factors which influence the attitude, perceived subjective norms, and perceived behavior control towards male involvement in maternal services utilization.

**Table 1:** Factor analysis

Factor analysis	Attitude	Subjective norms	Perceived behavior control
Initial questions analyzed	25	25	25
Initial Eigen values (before extraction)	Comp.1 = 36.072%; Comp. 2 = 14.290%	Comp.1 = 34.452%; Comp.2 = 18.777%	Comp. 1 = 37.207%; Comp. 2 = 15.216%
Final questions Question with component matrix > 0.3	15	15	16
Initial Eigen values (after extraction)	59.98%	57.26%	58.06
KMO measure	0.726	0.782	0.623
Mean	0	0	0

Positive = above mean; Negative = below mean

There were 25 different responses from five questions formulated based on the theory of planned behavior change for each predictor of intention. The responses were subjected to factor analysis and 15 statements of attitude and perceived subjective norms and 16 perceived behavior control had sample adequacy to test the three predictors of intention.

### Validity and reliability

To ensure the validity of the tool, a pilot study was conducted to assess the accuracy of the data collection tools. A Cronbach’s Alpha was conducted to establish the reliability of the tool. The Cronbach’s Alpha for attitude towards male involvement was 0.947, Cronbach’s Alpha for perceived subjective norms was 0.948 and a Cronbach’s Alpha for perceived behavior control was 0.938.

## Results

### Socio-demographic characteristics

The study enrolled 546 pregnant women at a turnover rate of 100%. The sample consisted of 546 pregnant women. The mean age was 25.57 years (sd = 6.810). The majority of the pregnant women were married (390, 71.4%), monogamous (469, 85.9%), live on less than 1 dollar per day (382, 70.0%), and receive their basic obstetric care services from dispensaries (452, 82.8). Ninety-five percent of the respondents had attained primary level education or less (Table 2).

**Table 2:** Socio-Demographic Characteristics of Respondents (*n* = 546)

Character	Female	Percents (%)
**Age (years)**		
Less than 20	167	30.6
20 to 25	156	28.6
26 to 30	105	19.2
31 to 35	55	10.1
36 and above	63	11.5
**Age at Marriage (years)**		
Less than 18	395	72.3
19 to 24	147	26.9
25 and above	4	0.7
**Ethnic group**		
Fipa	322	59.0
Mambwe	120	22.0
Others	104	19.0
**Marital status**		
Cohabit	156	28.6
Married	390	71.4
**Education level**		
Non-formal	230	42.1
Primary School	299	54.8
Secondary school or Higher	17	3.1
**Income per day**		
Less than 1 dollar	399	73.1
More than 1 dollar	147	26.9
**Own radio**		
Yes	253	46.3
No	293	53.7
**Own mobile phone**		
Yes	69	12.6
No	477	87.4
**Covered by Health Insurance**		
Yes	177	32.4
No	369	67.6
**Health facility**		
Dispensary	452	82.8
Health centre	94	17.2
**Distance to health facility (Km)**		
Less than 1	258	47.3
1 to 5	233	42.7
More than 5	55	10.1

Predictors of attitudes, subjective norms, and perceived behavior control towards male involvement in maternal services utilization.

### Predictor of attitude towards male involvement

The variables which portrayed a significant relationship with attitudes towards male engagement in maternal services utilization were age at marriage (*p* < 0.001), education status (*p <* 0.001), ethnic group (*p <* 0.001), economic status (*p* < 0.05), and owning a mobile phone (*p* < 0.001) (Table 3).

**Table 3:** The relationship between pregnant women’s characteristic and attitudes towards male involvement in maternal services utilization

Variables	Negative attitude		Positive attitude		X^2^	*p*-value
	Frequency	Percent (%)	Frequency	Percent (%)		
*Group*						
Intervention	116	21.2	66	12.1		
Control	200	36.6	164	30	3.846	0.05
*Age group*						
Less than 20	63	11.50	38	7.00		
21 to 25	94	17.20	60	11.00		
26 to 30	65	11.90	48	8.80	5.357	0.253
31 to 35	35	6.40	40	7.30		
36+	59	10.80	44	8.10		
*Age at marriage*						
Less than 18	158	28.90	76	13.90		
19 to 24	129	23.60	119	21.80	16.566	< 0.001
25 = +	29	5.30	35	6.40		
*Education status*						
No formal	136	24.9	58	10.60		
Primary school	166	30.40	158	28.90	18.471	< 0.001
Secondary school or higher	14	2.60	14	2.60		
*Ethnic group*						
Fipa	198	36.30	141	25.80		
Mambwe	41	7.50	71	13.00	41.752	< 0.001
Others	77	14.10	18	3.30		
*Economic status*						
Less than one dollar per day	234	42.90	151	27.70		
At least one dollar per day	82	15.00	79	14.50	4.516	0.034
*Own mobile phone*						
Yes	76	13.90	92	16.80		
No	240	44.00	138	25.30	15.896	< 0.001
*Walking distance*						
Less than 1 km	151	27.70	109	20.00		
1 to 5 km	133	24.40	97	17.80	0.017	0.992
More than 5 km	32	5.90	24	4.40		
*Ever heard about birth preparedness*						
Yes	252	46.20	196	35.90	2.705	0.100
No	64	11.70	34	6.20		

After adjusting for the confounders the factors which influence attitude towards male involvement in maternal services utilization among pregnant women were age at marriage [19 to 24 years, (AOR = 1.568 at 95% CI 1.044–2.353, *p* < 0.05), more than 24 years AOR = 2.15 at 95% CI = 1.150–1.159, *p <* 0.05)], education status [primary school AOR = 1.713 at 95%CI = 1.137–2.58, *p* = 0.01], ethnic group [Mambwe (AOR = 2.743 at 95% CI = 1.726–4.359, *p* < 0.001), Others (AOR = 0.425 at 95%CI = 0.235–0.768, *p* < 0.01)] and economic status [earning at least one dollar per day (AOR = 1.547 at 95%CI = 1.026–2.332, *p <* 0.05)] Table 4.

**Table 4:** Predictors of attitude towards male involvement among pregnant women and their male partners

Variables	AOR	95% CI		*p*-value
		Lower	Upper	
*Age at marriage*				
Less than 18	1			
19 to 24	1.568	1.044	2.353	0.03
25 = +	2.15	1.159	3.989	0.015
*Education status*				
No formal	1			
Primary school	1.713	1.137	2.58	0.01
Secondary school or higher	1.78	0.731	4.336	0.204
*Ethnic group*				
Fipa	1			
Mambwe	2.743	1.726	4.359	< 0.001
Others	0.425	0.235	0.768	0.005
*Economic status*				
Less than one dollar per day	1			
At least one dollar per day	1.547	1.026	2.332	0.037
*Own mobile phone*				
No	1			
Yes	1.283	0.837	1.965	0.253

### Predictor of subjective norms towards male involvement in maternal services utilization

The variables which showed a significant relationship with subjective norms towards male involvement in maternal services utilization among pregnant women were age at marriage (*p* < 0.001), education status (*p <* 0.01), ethnic group (*p <* 0.001), owning a mobile phone (*p* = 0.001) and having the intention to be accompanied by a male partner (*p <* 0.001) Table 5.

**Table 5:** The relationship between pregnant women’s characteristic and subjective norms towards male involvement

Variables	Negative subjective norms		Positive subjective norms		X^2^	*p*-value
	Frequency	Percent (%)	Frequency	Percent (%)		
*Group*						
Intervention	107	19.6	75	13.7		
Control	202	37	162	29.7	0.537	0.464
*Age group*						
Less than 20	57	10.40	44	8.10		
21 to 25	93	17.00	61	11.20		
26 to 30	60	11.00	53	9.70	4.598	0.331
31 to 35	36	6.60	39	7.10		
36+	63	11.50	40	7.30		
*Age at marriage*						
Less than 18	154	28.20	80	14.70		
19 to 24	128	23.40	120	22.00	16.006	< 0.001
25 = +	27	4.90	37	6.80		
*Education status*						
No formal	128	23.40	66	12.10		
Primary school	169	31.00	155	28.40	11.7	0.003
Secondary school or higher	12	2.20	16	2.90		
*Ethnic group*						
Fipa	195	35.70	144	26.40		
Mambwe	44	8.10	68	12.50	25.073	< 0.001
Others	70	12.80	25	237		
*Economic status*						
Less than one dollar per day	227	41.60	158	28.90		
At least one	82	15.00	79	14.50	2.979	0.084
*Own mobile phone*						
Yes	77	14.10	91	16.70		
No	232	42.50	146	26.70	11.437	0.001
*Would you like to accompany your partner to childbirth?*						
No	103	18.90	40	7.30		
Yes	206	37.70	197	36.10	18.788	< 0.001

After adjusting for the confounder, the predictors of subjective norms towards male involvement among pregnant women were age at marriage [19 to 24 years, (AOR = 1.447 at 95%CI = 0.970–2.159, *p <* 0.05), more than 24 years, (AOR = 2.331 at 95%CI = 1.261–4.308, *p <* 0.01), ethnic group [Mambwe, AOR = 2.278 at 95% CI =1.444–3.596, *p* < 0.001] Table 6.

**Table 6:** Predictors of subjective norms towards male involvement among pregnant women and their male partners

Variables	AOR	95% CI		*p*-value
		Lower	Upper	
*Age at marriage*				
Less than 18	1			
19 to 24	1.447	0.97	2.159	0.07
25 = +	2.331	1.261	4.308	0.007
*Education status*				
No formal	1			
Primary school	1.344	0.902	2.003	0.147
Secondary school or higher	1.761	0.736	4.211	0.203
*Ethnic group*				
Fipa	1			
Mambwe	2.278	1.444	3.596	< 0.001
Others	0.742	0.428	1.284	0.286
*Would you like to accompany your partner to childbirth?*				
No	1			
Yes	1.827	1.171	2.849	0.008
*Own mobile phone*				
No	1			
Yes	1.254	0.831	1.891	0.28

### Predictor of perceived behavior control towards male involvement in maternal services utilization

Variables which showed a significant relationship with perceived behavior control among pregnant women were age at marriage (*p <* 0.001), education status (0.01), ethnic group (0.001), own mobile phone (*p* = 0.001) and having the intention to be accompanied during childbirth (*p <* 0.001) Table 7.

**Table 7:** The relationship between pregnant women’s characteristic and perceived behavior control towards male involvement

Variables	Negative perceived		Positive perceived		X^2^	*p*-value
	Frequency	Percent (%)	Frequency	Percent (%)		
*Group*						
Intervention	107	19.6	75	13.7		
Control	202	37	162	29.7	0.537	0.464
*Age group*						
Less than 20	57	10.40	44	8.10		
21 to 25	93	17.00	61	11.20		
26 to 30	60	11.00	53	9.70	4.598	0.331
31 to 35	36	6.60	39	7.10		
36+	63	11.50	40	7.30		
*Age at marriage*						
Less than 18	154	28.20	80	14.70		
19 to 24	128	23.40	120	22.00	16.006	< 0.001
25 = +	27	4.90	37	6.80		
*Education status*						
No formal	128	23.40	66	12.10		
Primary school	169	31.00	155	28.40	11.7	0.003
Secondary school or higher	12	2.20	16	2.90		
*Ethnic group*						
Fipa	195	35.70	144	26.40		
Mambwe	44	8.10	68	12.50	25.073	< 0.001
Others	70	12.80	25	4.60		
*Economic status*						
Less than one dollar per day	227	41.60	158	28.90	2.979	0.084
At least one dollar per day	82	15.00	79	14.50		
*Own mobile phone*						
Yes	77	14.10	91	16.70		
No	232	42.50	146	26.70	11.437	0.001
*Would you like to accompany your partner to childbirth?*						
No	103	18.90	40	7.30	18.788	< 0.001
Yes	206	37.70	197	36.10		

After adjusting for confounders, the factors associated with confidence to involve their male partners in maternal services utilization were age at marriage [more than 24 years AOR = 2.331 at 95%CI = 1.261–4.308,*p* < 0.01], ethnic groups [Mambwe AOR = 2.278 at 95%CI = 1.444–3.596, *p* < 0.0001] and having the intention to be accompanied by their male partners AOR = 1.827 at 95%CI = 1.171–2.849,*p <* 0.01 (Table 8).

**Table 8:** Predictors of perceived behavior control among pregnant women and their male partners

Pregnant women				
Variables	AOR	95% CI		*p*-value
		Lower	Upper	
*Age at marriage*				
Less than 18	1			
19 to 24	1.447	0.97	2.159	0.07
25 = +	2.331	1.261	4.308	0.007
*Education status*				
No formal	1			
Primary school	1.344	0.902	2.003	0.147
Secondary school or higher	1.761	0.736	4.211	0.203
*Ethnic group*				
Fipa	1			
Mambwe	2.278	1.444	3.596	< 0.001
Others	0.742	0.428	1.284	0.286
*Would you like to accompany your partner to childbirth?*				
No	1			
Yes	1.827	1.171	2.849	0.008
*Own mobile phone*				
No	1			
Yes	1.254	0.831	1.891	0.28

## Discussion

Male involvement in maternal services utilization has been recognized as an effective strategy for the improvement of birth outcomes [18]. Many studies have reported male involvement and factors which influence male involvement focusing on males themselves [8, 9, 19]. Pregnant women’s attitudes, subjective norms, and perceived behavior control towards male involvement in maternal health services is an important behavioral aspect which if well addressed has the potential to improve male involvement. A female partner may act as a barrier towards bringing men to pregnancy care and childbirth. Their attitude, perceived subjective norms, and perceived behavior control matters a lot in their intention to have their male partners with them in maternal services utilization [5].

The study found that majority of pregnant women had a negative attitude, perceived subjective norms, and perceived behavior control towards male involvement in maternal services utilization. Age at marriage predicted all three domains of intention, attitude, perceived subjective norms, and perceived behavior control. The attitude towards male involvement in maternal services utilization was also influenced by pregnant women’s level of education and her economic status. In addition to age at marriage, the perceived behavior control was also influenced by pregnant women’s intention to be accompanied by her male partner. These findings are discussed hereunder.

The high proportion of pregnant women with negative attitudes towards the involvement of male partners in maternal health services utilization could be rooted in cultural beliefs and traditions and customs [5, 19]. Traditions and customs in most African cultures have assigned the role of pregnancy care and childbirth to women (Antenatal women and their mother and mother in law). In with accordance to *Theory of Planned Behavior,* the attitude towards a certain behavior can be influenced by the belief an individual has on the behavior and the way an individual evaluates the outcome of the behavior [13]. When pregnant women evaluate the outcome of male partner’s involvement in maternal health services utilization to have no contribution to the desired outcome, their attitude disregards male involvement in maternal health services utilization. Innovative interventions are highly recommended in this low resource setting to sensitize pregnant women on the benefits of male involvement in maternal health services utilization.

Likewise, the majority of pregnant women had negative perceived subjective norms towards male involvement in maternal health services utilization. This means that majority perceived that their community disregarded the accompaniment of their male partners in maternal health services. This perception is also stemmed from community beliefs and traditional gender roles [5, 19]. It sends a signal that insisting pregnant women come with their male partners during maternal services utilization alone without addressing their norms may delay male involvement in maternal services in our context. The effect of societal pressure on male involvement in maternal services utilization may act as a barrier towards male involvement in maternal services utilization. Innovative interventions are recommended to sensitize the community on the benefits of male involvement in maternal services utilization.

Similarly, the majority of pregnant women had negative perceived behavior control towards male involvement in the utilization of maternal health services. They perceive that they cannot bring their male partners in maternal health services utilization. Based on the *Theory of Planned Behavior,* perceived behavior control is influenced by control beliefs and perceived power [13]. Perceived behavior control could be affected by the low socio-economic status of the study community where a male partner has to engage in work to earn money for family sustainability.

The study found that factors which influence pregnant women’s attitude towards male involvement were age at marriage, education status, and economic status. Pregnant women who were married at the elder age were more likely to have a positive attitude towards male involvement in maternal health services than those who were married at a younger age. The possible reason for this finding could be that women who were married at a younger age did not have the opportunity to be exposed to formal education as compared to those who were married at an older age. Exposure to formal education can dilute the cultural beliefs of a woman which may influence power relations between men and women [20].

Pregnant women who had primary education were 1.7 times more likely to have a positive attitude towards male involvement than pregnant women who had no formal education. The finding is in line with a previous study which has reported a direct relationship between education and male involvement in maternal services utilization [20].

The study further noted that pregnant women who earned at least one dollar per day were 1.5 times more likely to have a positive attitude towards male involvement than pregnant women who earned less than one dollar per day. This could be poor women are concerned about their husbands engaging in earning work to sustain their living rather than participating in pregnancy care. A similar finding is reported by a previous study which reported that family earning do influence male involvement [23].

Age at marriage predicted the perceived subjective norms towards male involvement in maternal services utilization. Women who were married at the elder age were more likely to have a positive perception of societal approval for male involvement than women who were married at a young age. This finding could be women who are married at a younger age are less likely to have exposure to other societal cultural practices as they grow in the same culture. Those married at the elder age could have exposure to both education and travels to different places.

The age at marriage also influenced the perceived behavior control towards male involvement in maternal services utilization. Similarly, pregnant women who were married at the elder age, perceived to be able to be accompanied by their male partners for maternal services utilization. This could be because pregnant women who were married at a younger age could have stronger cultural attachment than those married at an elder age.

It was also found that pregnant women with the intention to be accompanied by their male partners were more likely to have positive perceived behavior control than those without the intention to be accompanied by their male partners.

This study used baseline data from an intervention study where control and intervention were compared. The two samples were treated as one sample after comparing the outcome variables (attitudes, subjective norms and perceived behavior control) and found no significant difference existed between the two groups. Intervention group participants were matched with control in a ratio of one to two. Even though in both cases random sampling was employed, our analysis may have suffered bias from differences in sampling probabilities in the two groups. There is a chance that some group is over represented than the other so may limit the generalizability of findings. To minimize the effect of this limitation, the participants were matched (5 years age groups and parity). The study also included robust of background information (ethnicity, economic status, exposure to media, education level, covered with health insurance, religion) in the data collection tool and were included in the analysis to adjust for the confounders.

Both groups came from rural districts of Rukwa region. Because rural Rukwa districts share similar cultural and social economic status, our findings can be generalized within rural Rukwa and other rural settings within Tanzania with similar characteristics.

## Conclusion

The study indicated that aged women are more likely to have a positive attitude, subjective norms, and perceived behavior control towards male involvement in maternal services than young pregnant women. Pregnant women with primary education, who earned more than a dollar per day were more likely to have positive attitudes towards male involvement than their counterparts. The intention to attend maternal services with their male partners significantly influenced positively the perceived behavior control. The study recommends a community based interventional study to address the community beliefs and traditional gender roles in maternal services utilization to improve pregnant women’s attitudes, subjective norms, and perceived behavior control towards male involvement in maternal services utilization. Behavior theory integrated interventions to address deep-seated predictors of male involvement and health-seeking behaviors have not been well explored in the existing literature. To understand and address such factors there is a need for innovative high-impact interventions that utilize theories, to address modifiable predictors of intention to engage in a behavior (Attitude, subjective norms, and perceived behavior control. The findings from such studies can be useful in shaping antenatal care interventions such as male involvement in maternal services utilization.

## Supplementary information

**Additional file 1.**

**Additional file 2.**

## Data Availability

Data set (supplementary file 1) and the questionnaire (supplementary file 2) are uploaded as supplementary material.

## Abbreviations

AIDS: Acquired Immunodeficiency Syndrome
ANC: Antenatal Clinic
HIV: Human Immunodeficiency Virus
MoHCDEC: Ministry of Health, Community Development, Gender, Elderly and Children
NBS: National Bureau of Statistics
STIs: Sexually Transmitted Infections
TDHS-MIS: Tanzania Demographic and Health Survey and Malaria Indicator Survey
