# Supplementary material for: Exploring factors influencing pregnant Women’s attitudes, perceived subjective norms and perceived behavior control towards male involvement in maternal services utilization: a baseline findings from a community based interventional study from Rukwa, rural Tanzania
Source: BMC Pregnancy Childbirth. 2020 Oct 19;20:634. doi: 10.1186/s12884-020-03321-z (PMC7574556; doi:10.1186/s12884-020-03321-z)
Supplement: Supplementary file 2 — Additional file 2. [file 12884_2020_3321_MOESM2_ESM.doc]

**QUESTIONNAIRES**

**First questionnaire: Female Partners**

**Part A: Baseline Information**

**Please tick (√) the appropriate option**

| 1. Age in years in years_____________ | | 1. Age at marriage in years_________ 2. Phone number_________________ |
| --- | --- | --- |
| 1. Marital status 2. Married ( ) 3. Cohabited ( ) | | 1. Educational status 2. None ( ) 3. Primary level incomplete ( ) 4. Primary level complete ( ) 5. Secondary or higher ( ) |
| 1. Employment status 2. Employed ( ) 3. Unemployed ( ) | | 1. Religion 2. Christian ( ) 3. Muslims ( ) 4. Others specify……………………… |
| 1. Ethnic group 2. Fipa ( ) 3. Mambwe ( ) 4. Others specify……………………… | 1. Economic status 2. Use less than one dollar per day ( ) 3. Use more than one dollar per day ( ) | |
| 1. Do you own radio? 2. Yes ( ) 3. No ( ) | | 1. Do you own mobile phone? 2. Yes ( ) 3. No ( ) |
| 1. Characteristic of a nearby health facility 2. Dispensary ( ) 3. Health center ( ) 4. Hospital ( ) | | 1. What is the walking distance to a nearby health facility? 2. Less than one kilometer ( ) 3. One kilometer-5kilometers ( ) 4. More than five kilometers ( ) |
| 1. Are you covered with health Insurance (NHIF or CHF)? 2. Yes ( ) 3. No ( ) | | 1. Pregnancy history 2. Primigravida ( ) 3. Para 1-Para 4 ( ) 4. Para 5+ ( ) |
| 1. Age at 1^st^ delivery (years)___________ | | |
| 1. Current pregnancy characteristics 2. Planned ( ) 3. Unplanned ( ) | | 1. Did you have prior pre-term delivery? 2. Yes ( ) 3. No ( ) 4. Not applicable ( ) |
| 1. Did you have a prior C-section? 2. Yes ( ) b) No ( ) c) Not applicable ( ) 3. Have you ever heard the term “birth preparedness”? 4. Yes ( ) b) No ( ) 5. Where did you hear about birth preparedness? 6. From health worker ( ) 7. From the media ( ) 8. From a family member ( ) 9. Others specify_________________________________________ | | |

**Part B: Female partners behavior intentions towards male involvement in maternal services utilization**

Many questions in this survey make use of rating scales with 5 places; you are to tick (√) the box that best describes your opinion where SA= strongly agree, A= agree, N= Neutral, D= disagree and SD = strongly disagree

| **Component** | **SA(5)** | **A (4)** | **N (3)** | **D (2)** | **SD(1)** |
| --- | --- | --- | --- | --- | --- |
| **B: Male involvement** |  |  |  |  |  |
| ***Attitude towards male involvement*** |  |  |  |  |  |
| 1. If my husband participate in setting aside some funds and equipments which will be used in case of emergency or during delivery is doing a good thing which is beneficial |  |  |  |  |  |
| 1. If my husband participate in preparation of transport which will be used in case of emergency or during childbirth is doing a good which is beneficial |  |  |  |  |  |
| 1. If my husband participate in identification of skilled attendant is doing a good thing which is beneficial |  |  |  |  |  |
| 1. If my husband accompany me during antenatal clinics is doing a good thing which is beneficial |  |  |  |  |  |
| 1. If my husband test for HIV with me during pregnancy is doing a good thing which is beneficial |  |  |  |  |  |
| 1. If my husband test for syphilis with me during antenatal clinics he is doing a good thing which is beneficial |  |  |  |  |  |
| 1. If my husband accompany me during childbirth he is doing a good thing which is beneficial |  |  |  |  |  |
| 1. If my husband accompany me for postnatal checkups he is doing a good thing which is beneficial |  |  |  |  |  |
| ***Assessment of subjective norms*** |  |  |  |  |  |
| 1. Important people to me think my husband has to participate in setting aside funds and equipments to be used in case of emergency or during childbirth. |  |  |  |  |  |
| 1. Important people to me think my husband has participate in preparation of transport to be used in case of emergency or during childbirth |  |  |  |  |  |
| 1. Important people to me think my husband should participate in identification of skilled birth attendant |  |  |  |  |  |
| 1. Important people to me think my husband has to accompany me during antenatal clinics |  |  |  |  |  |
| 1. Important people to me think my husband has to test for HIV with me during antenatal visits |  |  |  |  |  |
| 1. Important people to me think my husband has to screen for syphilis with me during antenatal visit |  |  |  |  |  |
| 1. Important people to me think my husband has to accompany me during childbirth |  |  |  |  |  |
| 1. Important people to me think my husband has to accompany me during postnatal checkups |  |  |  |  |  |
| ***Perceived Behavioral Control*** |  |  |  |  |  |
| 1. For my husband to participate in setting aside funds and equipments to be used in case of emergency or during childbirth is trouble free and he can do it |  |  |  |  |  |
| 1. For my husband to participate in preparation of transport to be used in case of emergency or during childbirth is simple and he can do it |  |  |  |  |  |
| 1. For my husband to participate in identification of skilled birth attendant is trouble free and he can do it |  |  |  |  |  |
| 1. For my husband to accompany me during antenatal clinics is simple and he can do it |  |  |  |  |  |
| 1. For my husband to test for HIV/AIDS with me during antenatal visits is trouble free and he can do it |  |  |  |  |  |
| 1. For my husband to test for syphilis with me during antenatal clinics is simple and he can do it |  |  |  |  |  |
| 1. For my husband to accompany me during labor and childbirth is simple and he can do it. |  |  |  |  |  |
| 1. For my husband to accompany me during postnatal checkup is trouble free and he can do it |  |  |  |  |  |
